# Evaluation of Electrolytically-Generated Hypochlorous Acid (‘Electrolyzed Water’) for Sanitation of Meat and Meat-Contact Surfaces

**DOI:** 10.3390/foods5020042

**Published:** 2016-06-13

**Authors:** Shawnna Veasey, Peter M. Muriana

**Affiliations:** 1 Department of Animal Science, Monroe Street, Oklahoma State University, Stillwater, OK 74078, USA; s_veasey@outlook.com; 2 Robert M. Kerr Food & Agricultural Products Centre, 109 FAPC Building, Monroe Street, Oklahoma State University, Stillwater, OK 74078-6055, USA

**Keywords:** electrolyzed water, hypochlorous acid, sanitation, biofilm, *Listeria*

## Abstract

‘Electrolyzed water’ generators are readily available in the food industry as a renewable source of hypochlorous acid that eliminates the need for workers to handle hazardous hypochlorite concentrates. We applied electrolyzed water (EW) directly to multi-strain cocktails of *Listeria monocytogenes, E. coli* O157:H7, and *Salmonella* sp. at 250 ppm free available chlorine (FAC) and achieved greater than 6-log reductions in 2 min. Lower EW values were examined as antimicrobial interventions for fresh meat (beef carcasses), processed meats (frankfurters), and food contact surfaces (slicing blades). Little or no reduction relative to controls was observed when generic *E. coli*-inoculated beef carcasses or *L. monocytogenes*-inoculated frankfurters were showered with EW. Spray application of EW (25 and 250-ppm FAC) onto *L. monocytogenes*-inoculated slicing blades showed that greater reductions were obtained with ‘clean’ (3.6 and 5.7-log reduction) *vs.* ‘dirty’ (0.6 and 3.3-log reduction) slicing blades, respectively. Trials with *L. monocytogenes*-inoculated protein-EW solutions demonstrated that protein content as low as 0.1% is capable of eliminating FAC, reducing antimicrobial activity against *L. monocytogenes*. EW appears better positioned as a surface sanitizer with minimal organic material that can otherwise act as an effective reducing agent to the oxidizing solution rendering it ineffective.

## 1. Introduction

The safety of fresh and processed meat products is no longer dependent solely on the sanitary practices of processors. The application of post-process lethality steps and/or antimicrobial ingredients or surface treatments have recently been incentivized and enhanced by regulatory authorities to promote safety and reduce risk from pathogenic bacteria during the manufacture of ready-to-eat (RTE) meat products [1]. Antimicrobials, such as organic acids and sodium hypochlorite, are commonly used antimicrobial agents in the fresh meat and poultry industries [2,3,4]. The processed meat industry commonly uses lactate and diacetate that have been shown to be particularly effective when used together against foodborne pathogens such as *Listeria monocytogenes* [5,6,7]. The U.S. Department of Agriculture’s Food Safety and Inspection Service (USDA-FSIS) ‘safe and suitable’ ingredient list includes ‘electrolytically generated hypochlorous acid’ [8].

Electrolytically generated hypochlorous acid is also generally referred to as ‘electrolyzed water (EW)’ or ‘electrochemically activated (ECA) water’ within the processing industry. Hypochlorous acid (HOCl) is the acidic equilibrium chemical variant of hypochlorite (OCl^−^) that exists as the predominant species below pH 6.6–6.8 and is considered more reactive than hypochlorite, although both are strong oxidants [9]. It is allowed for use in the fresh meat and poultry processing industries. Although hypochlorous acid can be generated from hypochlorite by pH-adjustment, automated electrolysis systems have been deployed by large commercial users and smaller systems are making their way into the facilities of smaller processors. These systems use electrolytic flow cells whereby streams are eluted from the anode or cathode regions to provide solutions often referred to as ‘anolyte’ or ‘catholyte’, respectively. The acidic anolyte solution contains the hypochlorous acid which is a very fast acting antimicrobial agent (oxidant) that destroys bacteria and other microorganisms in a very short period of time and has been shown to be an effective antimicrobial [10,11,12,13].

Hypochlorous acid has long been known to be detrimental to bacterial cells resulting from destructive oxidation of membrane bound complexes involved in ATP generation and maintenance of cellular electrical charge [12]. Studies have shown EW to be an effective sanitizer in several ways. It has been shown to be bactericidal to *E. coli* O157:H7, *L. monocytogenes*, *Salmonella* Enteritidis, and *S.* Typhimurium [14,15,16,17]. When used on contact surfaces, EW was shown to reduce biofilm formation and bacterial contamination [14,18,19,20]. With recent outbreaks of foodborne illness associated with leafy green vegetables, it is important to note that electrolyzed water can be used as a sanitizer on vegetables [11,21,22,23,24,25,26,27] and as a sanitizer for poultry carcasses and shell eggs [28,29,30].

Recent advances in the manufacture of long life, flow-through electrolytic cells, the widespread use of hypochlorite, and constant concern for problems with foodborne pathogens may account for the resurgence of new, self-contained electrolytic generators that have gained additional popularity in food/meat processing facilities. Although there are concerns for the generation of potentially carcinogenic byproducts when food or drinking water is contacted with reactive chlorine [31], they are allowed with limitations for use in reducing microbial contamination of foods. In this paper we examine the application of EW (hypochlorous acid) on raw and processed meats *vs.* food contact or equipment surfaces and provide data suggesting that EW may be better positioned as an environmental surface sanitizer than as a meat-contact sanitizer.

## 2. Materials and Methods

### 2.1. Bacterial Strains

Strains of *L. monocytogenes* used in this study included four that are moderately adherent to abiotic surfaces [*L.* monocytogenes 39-2 (retail hotdog isolate), V7-2 (serotype ½ a, milk isolate), 383-2 (ground beef isolate), and Scott A-2 (serotype 4b, clinical isolate)] and four that are strongly adherent to abiotic surfaces [*L. monocytogenes* 99-38 (ground beef isolate), CW62 (retail frankfurter isolate), CW50 (retail frankfurter isolate), and CW77 (retail frankfurter isolate)] [32]. Two generic *E. coli* strains (ATCC 51739 and ATCC 895) to inoculate beef carcasses, five pathogenic strains of *E. coli* O157:H7 [55(2)-AC1, 299(2)-AB3, 237(2)-AC1, 131(2)-AC1, and 114(2)-AC1] and two strains of *Salmonella* Enteritidis (CDC H3527 and CDC H3502) were also used in experiments with EW solutions. All bacteria were transferred from frozen stocks at −75 °C into sterile tryptic soy broth (TSB; Becton-Dickinson, Franklin Lakes, NJ, USA) tubes at a 1:100 dilution, incubated overnight at 30 °C, and then re-transferred for 18–20 h at 30 °C before use. Strains of *L. monocytogenes* used in this study were resistant to both streptomycin (100 μg/mL; Sigma-Aldrich, St. Louis, MO, USA) and rifamycin S/V (10 μg/mL; Sigma-Aldrich). *E. coli* strains were resistant to both novobiocin and streptomycin (100 μg/mL; Sigma-Aldrich). Recovery of inoculated organisms from non-sterile food products on media containing two antibiotics to which they were resistant excluded the recovery of indigenous bacteria; test platings with un-inoculated samples in the various trials we performed confirmed the adequacy of this approach. Bacteria tested in EW trials were first centrifuged (10 min, 10 °C, 6000 rpm) and then resuspended in sterile physiological saline (0.85% NaCl) to eliminate the reducing effects of growth medium proteins on hypochlorous acid. Mixtures of specific bacterial genera for various trials were then made by mixing equal proportions of individually resuspended bacteria.

### 2.2. Electrolyzed Water Generation

**Figure 1:**
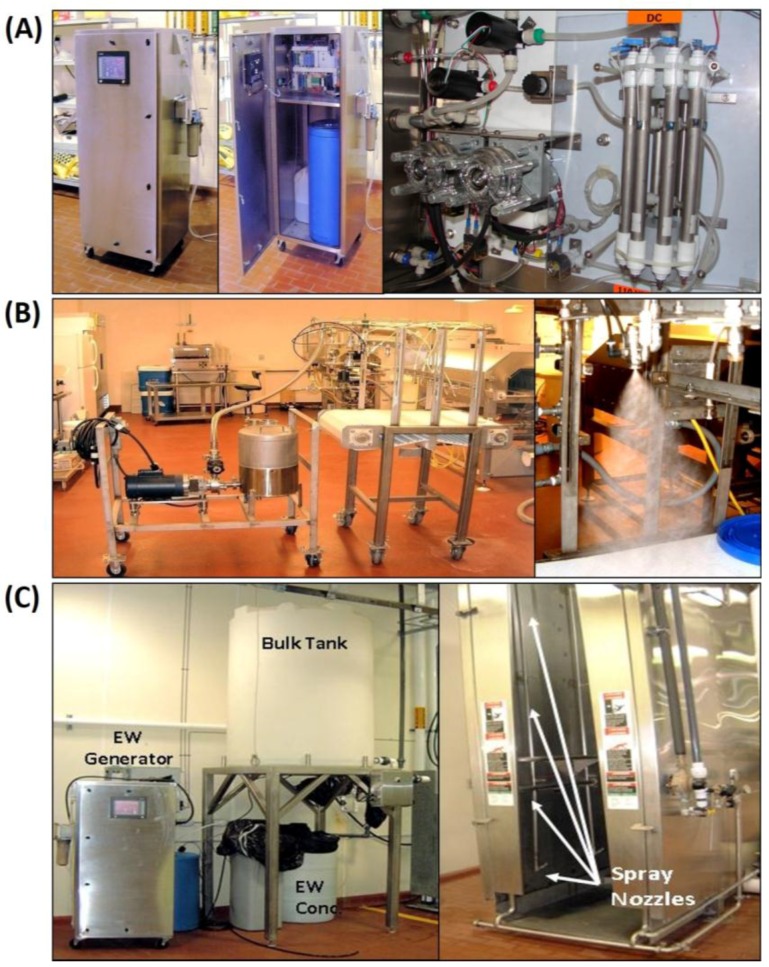
Major equipment used in this study. **Panel A**, electrolyzed water generator and electrolysis cells, provided by Unitherm Foodsystems (Bristow, OK, USA) and originally manufactured by Integrated Environmental Technologies (Little River, SC, USA); **Panel B**, pump, solution reservoir, and spray manifold for liquid or air-assisted (mist) spraying of liquid solutions; **Panel C**, electrolyzed water generator (EW), concentrate tank, and bulk tank setup (left) that was plumbed into a Frigoscandia Carcass Pasteurizer system for spraying full size carcasses.

Electrolyzed water was generated using an EcaFlo 080 EW generator (Figure 1A; Integrated Environmental Technologies, Inc, Little River, SC, USA) and supplied to Oklahoma State University by SanAquel LLC (Unitherm Food Systems Inc., Bristow, OK, USA). Electrolyzed water was generated at 5 Amps with 23% brine injection and at a pH of approximately 6.5 according to the manufacturer’s instructions (Figure 1A). On the day of the experiment, EW was manufactured and diluted to the desired free available chlorine (FAC) level using distilled water. Certain experiments required EW solutions at alternate pH levels which were modified by adjusting the proportion of acidic anode solution mixing with alkaline cathode solution. Proper cleaning and maintenance (acid flushing of the electrolysis chamber and tubes) were performed according to the manufacturer’s instructions.

### 2.3. Water Analysis

In all studies, whether reported or not, each treatment solution was analyzed for pH, oxidation-reduction potential (ORP), conductivity, total chlorine (TC), and free available chlorine (FAC). The pH and ORP were measured using an Oakton pH 110 (Oakton Instruments, Vernon Hills, IL, USA) combination meter according to the manufacturer’s directions. An Oakton Con 6 m was used to measure the conductivity according the manufacturer’s directions. Total chlorine was analyzed using the Hach (Loveland, CO, USA) digital titrator iodometric method (Method # 8209). Free available chlorine measurements were taken using the Hach digital titrator DPD-FEAS method (Method # 8210).

### 2.4. Effect of Electrolyzed Water on Bacterial Pathogens

The viability of *L. monocytogenes, E. coli* O157:H7*,* and *Salmonella* Enteritidis, in saline solution, was tested by addition to EW solution (250 ppm FAC) prior to subsequent dilution and plating. Samples were retrieved at 2 min intervals for 10 min. In another trial, a multi-strain mixture of *L. monocytogenes* was added to EW to result in final FAC levels of 5, 25, 50, or 100 ppm (pH 6.4–6.6). Controls consisted of bacterial suspensions in 0.1% buffered peptone water (BPW) as well as tap water in addition to suspensions made in EW. Each culture-EW solution was mixed well for 10 to 15 s and then samples were immediately diluted in 0.1% BPW and plated onto tryptic soy agar (TSA) using an EddyJet (IUL Instruments, Cincinnati, OH, USA). Plates were incubated for 48 h at 30 °C and counted using a colony counter (IUL Countermat Flash 5.0, IUL Instruments, Plainview, NY, USA).

### 2.5. Spray Systems

Several spray systems were used in our experiments for frankfurters and beef carcasses (Figure 1B,C, respectively).

For treatment of frankfurters, a manufactured spray system was used consisting of a 5-gal reservoir tank and an FPX 702-100 centrifugal pump (Fristam, Middleton, WI, USA) that was regulated by an Allen-Bradley Pico 1760 controller (Rockwell Automation, Tulsa, OK, USA) for timed-spray increments. This setup was connected to an 80-psi air-atomizing spray nozzle (Spraying Systems Co., Wheaton, IL, USA) connected to our in-house air pressure line, and was adjusted to deliver only 200 mL/min depending on air pressure valve and pump restrictor settings (Figure 1B). Electrolyzed water was tested at different pH levels on frankfurters inoculated with *L. monocytogenes.* Frankfurters were manufactured in the Robert M. Kerr Food and Agricultural Products Center (FAPC) meat pilot plant without addition of antimicrobials. Frankfurters were dip-inoculated into a four-strain cocktail of strongly-adherent strains of *L. monocytogenes* in 0.1% BPW at approximately 10^8^ colony forming units per mL (CFU/mL), removed after 5 min, and placed at 5 °C for 15 min to allow for attachment. Frankfurters were placed in sterile baskets for spray treatment that allowed rinse liquids to be collected into sterile containers positioned below. Frankfurters were sprayed for 30 s with EW solutions at concentrations of 27–39 ppm FAC and set at different pH levels (pH 4.0, 5.0, and 6.0) to see if EW at a lower pH range results in any greater antimicrobial activity. Remaining viable bacteria were removed from the frankfurters by massaging 2 mL of 0.1% BPW around each sample, diluting in 0.1% BPW, and plating on TSA. Recovered spray solutions for each treatment were also plated. Plates were incubated for 48 h at 30 °C and enumerated with a colony counter.

For carcass spray treatment, an EcaFlo 080 EW generator was connected to a 50-gal concentrate tank that supplied a larger 500-gal working-stock storage tank which was connected by PVC pipe to 16 spray nozzles at different carcass heights in a Carcass Steam Pasteurizer system (Frigoscandia, Stockholm, Sweden; Figure 1C). Two Berkeley BPDH10-L self-priming centrifugal pumps were used: one to transfer EW concentrate to the working stock tank; the other to send working stock solution to the spray system. The carcass pasteurizer system in the animal abattoir in FAPC is generally used to steam-sanitize all animal carcasses after slaughter. The pasteurizer was modified so that EW solutions could be sprayed from storage tanks through the same steam pipes and 16 nozzles that would otherwise dispense steam to carcass surfaces (Figure 1C). This modification was used to determine EW’s efficacy on reducing surface microflora on beef carcasses of cattle that had been harvested in FAPC’s meat pilot plant abattoir facility. On each carcass half, a 20 × 10 cm^2^ area was marked using a sterile stainless steel frame and edible dye. The area was sponge-inoculated with a two-strain mixture of overnight culture of generic (non-pathogenic) *E. coli* diluted 1:10 with 0.1% BPW (*i.e.*, ~1 × 10^8^ CFU/mL). Carcass halves were held for 30 min after inoculation and prior to spraying to allow for bacterial attachment. Microbial swab sample platings using our antibiotic-selective media of un-inoculated carcass surfaces within the designated inoculation area were performed to insure the absence of background on our plating media. Carcass halves were then sprayed for 30 s in a carcass spray chamber (40–60 psi; 50 ppm FAC; 1 liter/min/nozzle). Residual spray solutions (runoff) were recovered by positioning a container below each carcass. A similar procedure was performed with carcasses using tap water as a control solution. Sterile sponges were then used to swab remaining viable bacteria from the inoculated area on each sample. Sponges were then placed in a sterile stomacher bag with 20 mL of 0.1% BPW, stomached for 2 min, and serial dilutions made with 0.1% BPW were spiral plated onto TSA using an EddyJet (IUL Instruments) spiral plater. Plates were incubated for 48 h at 30 °C and counted using an automated colony counter (IUL Countermat Flash 5.0, IUL Instruments).

Controls often consisted of inoculated (pretreatment) as well as water spray treatments (non-lethal) in comparison to EW spray treatments (lethal) in order to determine whether reduction of microbial counts was due to liquid displacement from inoculated surfaces rather than lethality from antimicrobial solutions.

### 2.6. Efficacy of Electrolyzed Water in the Presence of Various Protein Levels

The effect of known levels of organic material (*i.e.*, protein levels) was examined on the oxidizing capacity of hypochlorous acid and its subsequent ability to kill bacteria. Electrolyzed water was used at an FAC level of 55 ppm. The diluted EW solution was dispensed in 90 mL aliquots to which 10 mL of protein solution would be added, to result in a final concentration of ~49 ppm FAC. Gelatin (Sigma-Aldrich) solution was dissolved in distilled water at 1.0%, 0.5%, and 0.25%, and 10-mL volumes were added to the 90-mL EW samples to obtain final concentrations of 0.1%, 0.05%, and 0.025% protein, respectively. Controls were 90 mL aliquots of EW solutions to which 10 mL of distilled water (no protein) was added. Following the addition of soluble gelatin protein, each solution was tested for total chlorine and FAC. After 2 min of gelatin treatment, 9.9-mL aliquots of EW-protein solutions or BPW were dispensed into sterile test tubes containing a multiple-strain cocktail of washed cells of *L. monocytogenes* (0.1 mL). The BPW tube (non-lethal) represented the diluted bacterial mixture viable count (negative control) while the EW solution without added protein represented the positive control. The test tubes were vortexed for 30 s, diluted with 0.1% BPW, and spiral-plated using an EddyJet (IUL Instruments) on TSA, incubated for 48 h at 30 °C, and then counted using a colony counter (IUL Countermat Flash 5.0, IUL Instruments).

### 2.7. Efficacy of Electrolyzed Water on Contaminated Slicing Blades

The efficacy of EW to inhibit *L. monocytogenes* on 5 × 5 cm^2^ inoculated sections of stainless steel slicing blades was examined using clean and ‘dirty’ slicing blades. Clean blades were free of organic material prior to inoculation. ‘Dirty blades’ had been smeared with pieces of processed ham, to simulate the process of slicing processed meat, and then allowed to dry prior to inoculation. Each blade had been previously marked with a 5 × 5 cm^2^ template (25 cm^2^) using an indelible marker. Then, 100 µL of a four-strain cocktail of washed cells of strongly adherent strains of *L. monocytogenes* was spread with a gloved finger throughout the marked area. The inoculum was allowed to attach to the blades for 30 min at 5 °C before use. The blades were then placed in sterile baskets and received a 30-s pressurized spray (20–40 psi) treatment (Figure 1B). Deionized glass-distilled water was used for control rinses (*i.e.*, 0 ppm FAC) while EW solutions were used at 5-, 25-, and 250 ppm FAC. After spray treatment, remaining bacteria were removed from the blades using a sponge moistened with 25 mL of 0.1% BPW (*i.e.*, 1 mL ≈ 1 cm^2^). The sponge swab was placed back into a stomacher bag and stomached for two minutes on a medium setting. After serial dilutions in 0.1% BPW, each sample was spiral plated using an EddyJet (IUL Instruments) on TSA. Plates were incubated for 48 h at 30 °C then counted using a colony counter (IUL Countermat Flash 4.2, IUL Instruments).

### 2.8. Effect of EW on L. monocytogenes Biofilms

Biofilms of four strongly-adherent strains of *L. monocytogenes* (strains CW50, CW62, CW77, and 99–38) were made using CultureSlide chambers (Falcon, Becton-Dickinson, Bedford, MA, USA) as described previously [32]. After incubation, the eight-well plastic chambers were removed so that the bottom surface (*i.e.*, a microscope slide) possessed the attached biofilm. Biofilms made in CultureSlide chambers with the four strongly-adherent strains were washed with 0.1% BPW or with EW (250 ppm) for 2 min, and then processed for scanning electron microscopy (SEM) at the OSU Electron Microscopy Core Facility (Oklahoma State University, Stillwater, OK, USA).

### 2.9. Statistics

All trials were performed in triplicate replication and error bars represent the standard deviation of the mean. For most experiments, the results were analyzed using a one-way analysis of variance (ANOVA). A pairwise multiple comparison was then completed for each using the Holm-Sidak method. All statistical analyses were performed using Sigma Plot (Systat Software Inc, San Jose, CA, USA) at a *p*-value < 0.05.

## 3. Results and Discussion

### 3.1. Antimicrobial Activity of EW Solutions

The predominant active agent in EW is considered to be free available chlorine (FAC) that is present as hypochlorous acid when the solution is below pH 6.8, or as hypochlorite when it is above that pH level. Some companies prefer to use oxidation reduction potential (ORP) to measure EW solution strength because of the ease of using pocket ORP analyzers (similar to pocket pH meters), whereas a comparable portable FAC analyzer requires a more tedious chemical titration. However, both USDA and FDA stipulate that measurement of the FAC is the only factor that determines regulatory compliance, as ORP does not correlate to FAC.

**Figure 2:**
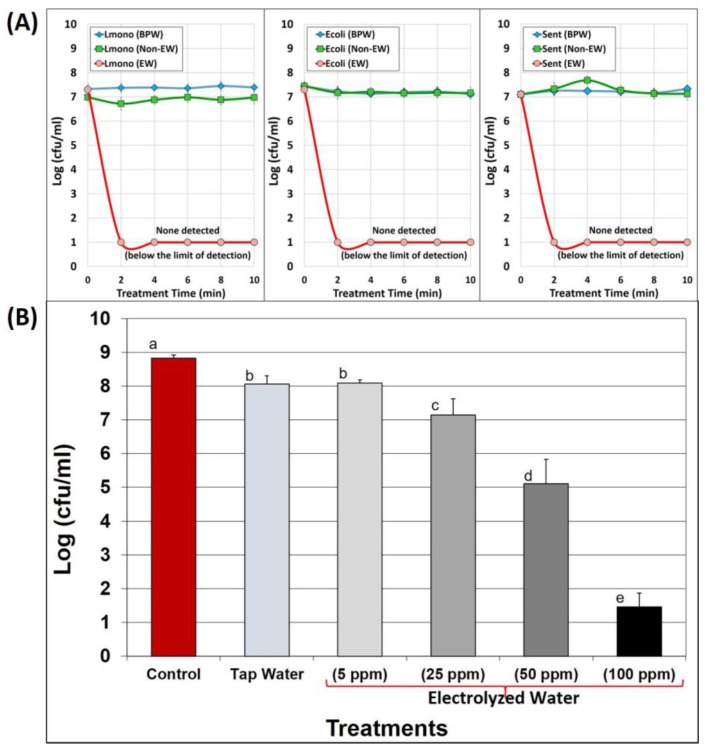
Effect of electrolyzed water (EW) on pathogenic bacteria. **Panel A**, effect of EW (250 ppm free Cl^−^) on mixtures of strains of *E. coli* O157:H7, *Salmonella* Enteritidis, or *Listeria monocytogenes* for 0, 2, 4, 6, 8, and 10 min at room temperature in 0.1% buffered peptone water (BPW), tap water/non-EW (NEW), or EW. The data points are the means of duplicate trials (error bars were not shown to prevent clutter); **Panel B**, four-strain mixture of washed cells of *L. monocytogenes* subjected to short treatment time (~15 s) with buffered peptone water (BPW), tap water, and EW (5-, 25-, 50-, and 100 ppm free available chlorine (FAC). All trials were performed in triplicate replication of paired samples, and data points represent the means. Treatments with the same lower case letter are not significantly different (*p* > 0.05); treatments with different lower case letters are significantly different (*p* < 0.05).

Electrolyzed water was tested on various pathogens of importance to the food industry, demonstrating that EW at higher FAC levels is effectively bactericidal to pathogens in solution. At 250 ppm FAC, no viable cells were recovered from trials with either *E. coli* O157:H7, *Salmonella* Enteritidis, or *L. monocytogenes*, generating >6-log reduction of these pathogens in solution (Figure 2A). We further examined the effectiveness of lower FAC levels on *L. monocytogenes* alone (Figure 2B). Electrolyzed water at 25, 50, and 100 ppm was effective in reducing *L. monocytogenes* by 1.67, 3.72, and 7.36 logs (CFU/mL), respectively, when compared to washed cells suspended in 0.1% buffered peptone water (BPW) controls (Figure 2B). However, EW at 5 ppm FAC was no more effective than tap water (*i.e.*, <1 log reduction). Although the US-EPA allows ≤4 ppm FAC in drinking water, the USDA-FSIS allows ≤5 ppm FAC in water used for meat processing, ≤20 ppm on beef subprimals, 20–50 ppm in various poultry processing/chill water applications, and ≤50 ppm on beef carcasses [8]. FAC levels as high as 200 ppm are allowed as sanitizers on food contact surfaces, although there would have to be sufficient time for drainage of residual liquid, or a post-application rinse with water, before food contact is allowed, as transfer of residual EW to the food would occur. This would constitute EW as a ‘direct food additive’ on RTE meats which is not currently allowed and would require a rinse with tap water prior to contact with RTE meats. The reduction observed with 5 ppm FAC was only permitted due to the removal of media proteins from bacterial cultures in using washed cells, as we have observed that the direct addition of cultures directly from media growth tubes does not show the same reduction at 5 ppm FAC as observed here (Figure 2B).

### 3.2. Application of EW to Raw Beef Carcasses

**Figure 3:**
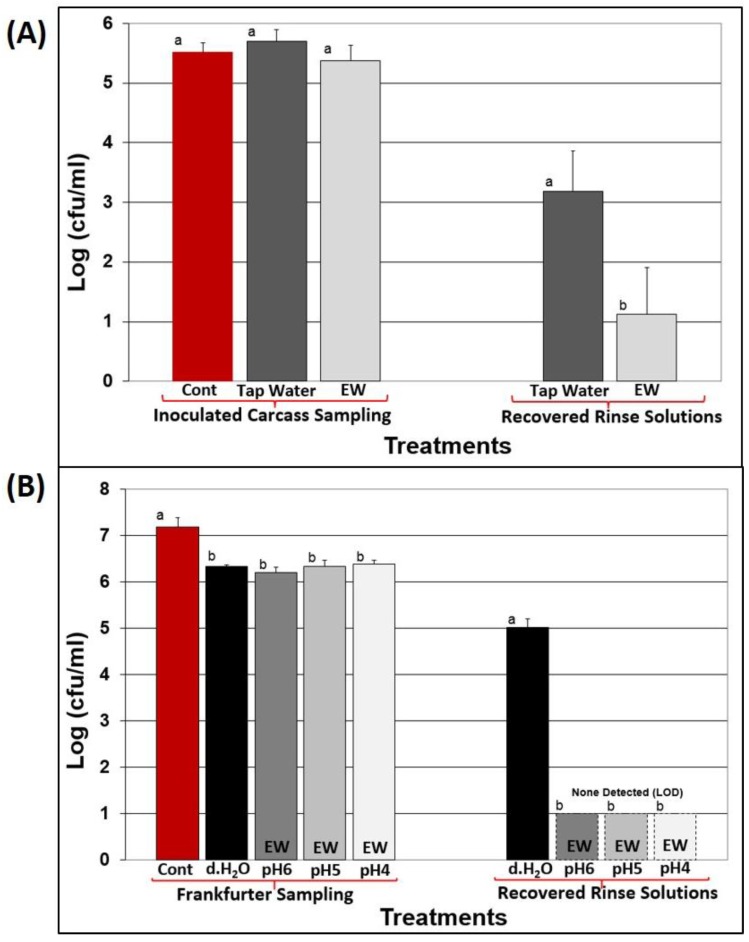
Electrolyzed water (EW) treatment of *E. coli*-inoculated beef carcasses and *Listeria monocytogenes*-inoculated frankfurters. **Panel A**, carcass halves inoculated with generic *E. coli* were sprayed for 30 s with either EW (50 ppm) or tap water using a modified Frigoscandia Carcass Steam Pasteurizer System in which the steam spray system was retrofitted to accommodate liquid solutions; **Panel B**, frankfurters inoculated with a four-strain cocktail of *L. monocytogenes* spray treated with an air-assisted sprayer (80 psi) for 30 s. Spray solutions were distilled water and EW at pH 4 (30 ppm), pH 5 (27 ppm), and pH 6 (39 ppm). In both sets of experiments, the rinse solutions were also recovered and plated. All trials were performed in triplicate replication, and data points represent the means. Within each treatment, means with the same lower case letters are not significantly different (*p* > 0.05); means with different lower case letters are significantly different (*p* < 0.05).

A Frigoscandia carcass surface steam pasteurizer was modified to allow the application of EW spray through 16 spray nozzles surrounding the hanging (half-) carcasses that would otherwise deliver steam for surface pasteurization (Figure 1C). Spray treatments using EW at the maximum level of FAC allowed (50 ppm FAC) were compared to spray treatments with tap water on beef carcasses inoculated with a mixture of generic *E. coli* strains (non-pathogenic). No significant differences in effect were observed between the two solutions (Figure 3A). EW was not effective in reducing levels of surface-inoculated generic *E. coli* on carcasses even with the aid of the multi-nozzle spray system despite prior laboratory trials with inoculated beef sections showing moderate levels of reduction (data not shown). This could be due to a greater degree of soluble protein running off the surface of freshly slaughtered beef carcasses. The rinse solutions (from the entire carcass) that were recovered and plated showed generic *E. coli* levels were significantly lower in the recovered EW solutions than when carcasses were rinsed with tap water (Figure 3A). One might have expected surface-inoculated bacteria to be readily killed by spray treatment of oxidizing solutions. The data suggests that the high organic load immediately beneath the inoculated areas (or soluble protein dripping over the inoculated cells) buffers the surface-inoculated cells from the hypochlorous acid, rendering it ineffective even though there is residual activity sufficient to kill bacteria in the recovered rinse solutions that is not observed with tap water. Our data showing the ineffectiveness of EW solutions in reducing microbial counts on raw beef corroborates the report by Fabrizio and Cutter [33] who showed no significant differences in raw pork bellies inoculated with *Salmonella* or *Listeria* when spray treated with acidic EW (~50 ppm FAC) in comparison to samples treated with distilled water.

### 3.3. Antimicrobial Treatments with RTE Meats

The potential use of EW as an antimicrobial for RTE meats was also examined. Although hypochlorous acid (or hypochlorite) is not allowed on RTE meats in the USA according to FDA and USDA regulations, it is used on RTE meats elsewhere in the world (*i.e.*, South Africa). In the USA, EW can be used on plastic-encased RTE products but not on permeable (cellulose) encased products in which it would still be considered to be subject to ‘food contact’. Electrolyzed water can also be used as a surface sanitizer on equipment surfaces if followed up with a spray of tap water prior to re-contact with RTE meats. In previous experiments, we used FAC concentrations that were allowed by the USDA-FSIS on raw beef. At the time of these experiments, EW was not recognized as a safe and suitable ingredient by the USDA-FSIS on RTE meats and therefore we based our FAC concentrations for the following experiments on those allowed for fresh meats.

In prior experiments, EW was used at approximately pH 6.5–6.7, pushing the equilibrium to the hypochlorous acid form (above ~pH 6.8, it should be in the hypochlorite form). During trials with *L. monocytogenes*-inoculated frankfurters, EW solutions at pH 6, pH 5, and pH 4 were tested to see if hypochlorous acid reactivity would be further enhanced at lower pH levels, even though EW at pH 4 may have a greater degree of dissolved Cl_2_ due to potential off-gassing at low pH levels (Figure 3B). We observed no significant difference in efficacy of *L. monocytogenes*-inoculated frankfurters sprayed with distilled water *versus* the various pH/EW solutions; however, there was a dramatic difference between the survival of *L. monocytogenes* in the recovered rinse treatments, whereby distilled water showed a 5-log CFU/mL level in recovered rinse solution in contrast to <1-log CFU/mL from all EW solutions tested (Figure 3B). The greater difference in viable organisms in the recovered rinse solutions from hotdogs *vs.* those from raw beef carcasses may be due to a greater degree of organic material from freshly slaughtered raw beef carcasses (soluble protein) than from a cooked RTE processed meat, whereby ingredient proteins components have been congealed by cooking and less soluble when rinsed.

Fabrizio and Cutter [34] also examined the effect of acidic and basic EW on *L. monocytogenes*-inoculated frankfurters, however, their comparisons were with treatments relative to inoculated but untreated controls. Without comparative water-spray controls, it is difficult to determine the true lethality of treatment, as one may simply be experiencing physical displacement of inoculated organisms by a non-lethal solution (*i.e.*, water) that would otherwise be perceived as microbial reduction due to lethality.

### 3.4. Antimicrobial Activity of EW on Slicing Blades

**Figure 4:**
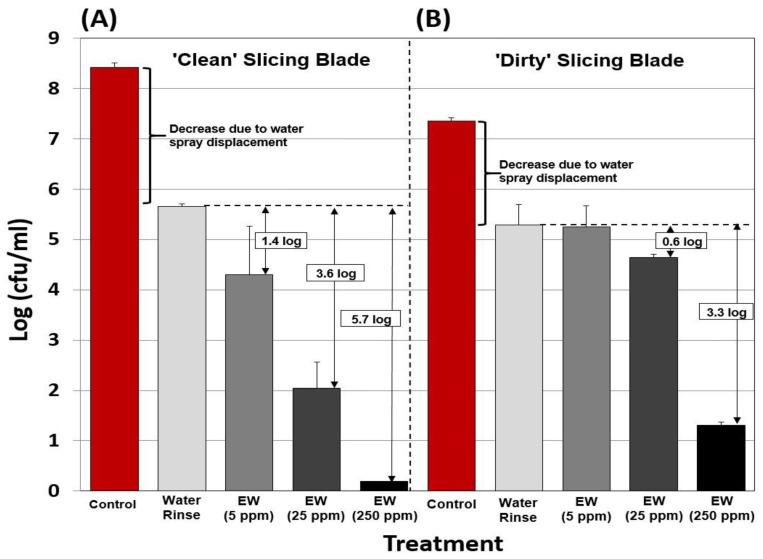
Efficacy of electrolyzed water (EW) *vs.* *L. monocytogenes* on stainless steel slicing blades. Clean (**panel A**) or dirty (**panel B**) sections (5 × 5 cm^2^) of stainless steel slicing blades were inoculated with *L. monocytogenes* and spray treated with water, 5-, 25-, or 250 ppm FAC of EW. Slicing blades were rendered ‘dirty’ by making several cuts through ready-to-eat (RTE) deli turkey breast to condition the blade with an organic load. All trials were performed in triplicate replication, and data points represent the means. Means with the same lower case letters are not significantly different (*p* > 0.05); means with different lower case letters are significantly different (*p* < 0.05).

*L. monocytogenes* is an important problem pathogen in RTE food handling/processing facilities because it can form biofilms on equipment surfaces, develop within harborage sites, and become a source of contamination. *L. monocytogenes* is a significant problem in such facilities because the resulting RTE foods are not cooked prior to consumption and therefore sanitation vigilance is paramount for facilities handling RTE meat products. A common food contact surface that has been involved in microbial contamination and is responsible for ‘touching’ vast quantities of food are stainless steel slicing blades for the manufacture of RTE meats, whether pre-sliced luncheon meats or spiral-sliced hams manufactured by meat processors, or from slicers found at supermarket delicatessens. The effect of EW was examined as a sanitizing rinse for stainless steel slicing blades by comparing the efficacy of water *vs.* EW at 5, 25, and 250 ppm FAC on both clean and ‘dirty’ blades (Figure 4). On clean blades, the impact of the spray rinse washed off a considerable level of the *L. monocytogenes* inoculum that was allowed to adhere for 30 min (Figure 4A). A 1.4-, 3.6-, and 5.7-log reduction was obtained for EW rinse treatments of 5, 25, and 250 ppm FAC, respectively, over and above that which was reduced by water displacement (Figure 4A). However, on ‘dirty’ blades pre-soiled before inoculation by slicing motions with a RTE processed ham product, the reduction of *L. monocytogenes* was limited to 0-, 0.64-, and 3.3 logs, respectively, with EW of 5, 25, and 250 ppm FAC (Figure 4B). The data demonstrates how the presence of underlying organic material significantly reduces the effectiveness of the hypochlorous acid in EW, especially at lower FAC levels, and is likely the reason for poor microbial reductions on applications with inoculated meat products (*i.e.*, high organic load). The hypochlorous acid is getting reduced by the organic material before it has a chance to interact with the bacteria that might be present.

### 3.5. Effect of EW in the Presence of Known Protein Levels

**Figure 5:**
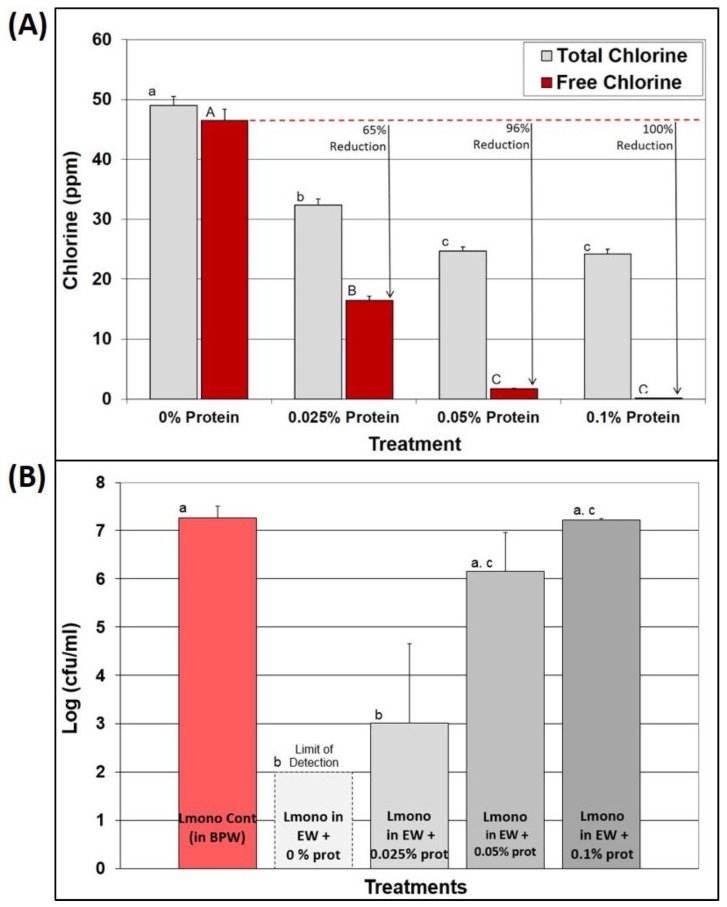
Effect of organic material on free available chlorine and antimicrobial effectiveness of protein-electrolyzed water (EW) solutions on *L. monocytogenes*. **Panel A**, EW (50 ppm) was mixed with fish gelatin for final protein concentrations of 0.025%, 0.05%, and 0.1%, and then free and total chlorine was determined; **Panel B**, EW (50 ppm) was mixed with gelatin from fish for final protein concentrations of 0.1%, 0.05%, and 0.025%. A four-strain cocktail of *L. monocytogenes* was then added, vortexed (30 s), and then plated. All trials were performed in triplicate replication, and data points represent the means. Means with the same lower- or upper-case letters are not significantly different (*p* > 0.05). Means with different lower- or upper-case letters are significantly different (*p* < 0.05).

A protein solution assay was used to further examine the effect of organic material on the residual FAC concentration of EW after a brief exposure as well as on antimicrobial efficacy before, and after, the addition of protein. Protein levels as low as 0.025% resulted in a 65% reduction of FAC levels in EW, a 96% reduction in FAC levels at 0.05% protein, and 100% elimination of FAC at 0.1% protein (Figure 5A). When *L. monocytogenes* was added to these EW/protein solutions, the antimicrobial capability of EW dramatically reduced thereby allowing greater survival of *L. monocytogenes*, and correlating to the reduction observed with FAC levels (Figure 5B). Even with dissolved protein levels as low as 0.1%, EW efficacy was rendered nil and had little or no effect on reducing *L. monocytogenes* in solution (Figure 5B). This also explains how 0.1% BPW (*i.e.*, sample dilutions) and/or cell culture suspended in growth media (protein) may reduce the oxidizing capacity of EW that might be added to it.

### 3.6. Effect of EW on L. monocytogenes Biofilms

**Figure 6:**
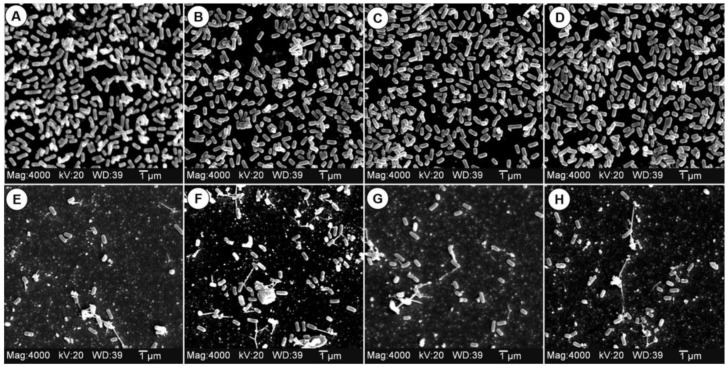
Scanning electron microscopy (SEM) of biofilms made with the four strongly-adherent strains of *L. monocytogenes* CW50 (**panel A**), CW62 (**B**), CW77 (**C**), and 99-38 (**D**) washed with either water (**panels A–D**) or with EW (250 ppm) for 2 min (**panels E–H**).

*L. monocytogenes* CW50, CW62, CW77, and 99-38 are strongly-adherent strains that are capable of forming effective biofilms on glass, plastic, stainless steel, and rubber surfaces (Figure 6A–D) [32]. Using eight-well culture chambers attached to microscope slides, we were able to establish biofilms on the bottom chamber surfaces (*i.e.*, microscope slide) such that multiple strains could readily be compared by electron microscopic analysis by removal of the plastic chamber. In short order (24–36 h), these strains can form biofilms that nearly form confluent monolayers on surfaces (Figure 6A–D). When surface biofilms were treated with 250 ppm FAC of EW, the field of view observed by SEM shows a debris field of lysed or dead cells and complements our results with EW as an effective antimicrobial on equipment or environmental surfaces that are relatively free of heavy organic loads that would otherwise react with hypochlorous acid. This could be an easy solution to eliminating young, immature biofilms that have not yet established a thick organic layer. Ayebah *et al.* [14] also used EW to obtain 4.3–5.2-log CFU reductions per 2 × 5-cm coupons with 48-h cultured biofilms of *L. monocytogenes*. The SEM data shown here also complements the transmission electron microscopy (TEM) images of Kiura *et al.* [35] with *Pseudomonas aeruginosa* that showed EW to cause changes in cellular morphology and bleb formation to implicate damage to the outer membrane of Gram-negative bacteria, leading to cellular destruction. Our data suggests similar destruction also occurs to the cell wall structure of Gram-positive bacteria (Figure 6).

## 4. Conclusions

Current regulations (in the USA) do not permit the use of EW on RTE meats, although it is allowed in other countries. However, it is allowed for use on fresh meat and poultry at levels from 20 ppm (retail cuts) to 50 ppm (carcasses) FAC [8]. Increasing the allowable levels of FAC may likely increase the effectiveness of EW on fresh meat products, however, use-level limitations are meant to limit the production of disinfection byproducts in chlorine-treated foods [31]. Hypochlorous acid, however, can still be used as a surface sanitizer at higher levels than those used on meats, but would have to either allow a period for it to drip off food contact surfaces, or be rinsed with potable water before food samples are applied to the surfaces (*i.e.*, conveyor belts). Electrolyzed water was also not effective when sprayed directly onto RTE meats, although sanitation of encased product surfaces may reduce the exposure of organic material to the hypochlorous acid. Hypochlorous acid is allowed for use as a surface sanitizer only on RTE meats that are encased in impermeable casing, and such applications are showing greater appeal as a sanitizing rinse on vacuum-package logs of RTE meats prior to unbagging for a slicing operation of pre-sliced luncheon meats.

At the levels examined, EW was not an effective antimicrobial when directly applied to meats, but rather as a sanitizer for processing environments, food contact surfaces, and product wrapped in impermeable cases where minimal organic material may be present. High organic loads on meat surfaces render hypochlorous acid ineffective as an antimicrobial intervention, despite the fact that bacteria were inoculated on the outermost surface. Considering our results with even dilute protein solutions (<0.1% protein) that quickly extinguished the oxidizing capacity of hypochlorous acid, it is not clear how others obtain activity in the presence of organic material (*i.e.*, solid meat). Special consideration should be given to the use of water control treatments for comparison with antimicrobial treatments that may be mistakenly perceived as microbial reduction. However, hypochlorous acid remains an effective surface sanitizer when faced with minimal organic loads, as might be found during routine post-detergent sanitation operations.

Based on the data presented herein on the sensitivity of oxidizing solutions (hypochlorous acid, ozone) to organic material, all food processors using these solutions as antimicrobial interventions should perform validated studies to confirm that they are performing as expected in their operations.

## Abbreviations

The following abbreviations are used in this manuscript:

MDPI: Multidisciplinary Digital Publishing Institute
EW: Electrolyzed water
FAC: Free available chlorine
RTE: Ready-to-eat
USDA: United States department of agriculture
FSIS: Food safety and inspection service
HOCL: Hypochlorous acid
PPM: Parts per million
OCL: Hypochlorite
ECA: Electrochemically activated
ATTC: American Type Tissue Collection
PSI: pounds per square inch
PVC: polyvinyl chloride
ORP: Oxidation reduction potential
BPW: Buffered peptone water
TSA: Tryptic soy agar
ANOVA: Analysis of variance
